# Development and Validity of an Intrapartum Self-Assessment Scale Aimed at Instilling Midwife-Led Care Competencies Used at Freestanding Midwifery Units

**DOI:** 10.3390/ijerph20031859

**Published:** 2023-01-19

**Authors:** Naomi Inoue, Yuko Nakao, Atsuko Yoshidome

**Affiliations:** School of Health Sciences, Kagoshima University, Kagoshima 890-8544, Japan

**Keywords:** midwife-led care, clinical competence, freestanding midwifery units, childbirth

## Abstract

Building experience in midwife-led care at freestanding midwifery units is needed to enhance assessment, technical, and care competencies specific to midwives. This study aimed to develop a self-assessment scale for midwifery practice competency based on the characteristics of midwife-led care practices in freestanding midwifery units. This study was conducted at 65 childbirth facilities in Japan between September 2017 and March 2018. The items on the scale were developed based on a literature review, discussion at a professional meeting, and a preliminary survey conducted at two timepoints. The validity and reproducibility of the scale were evaluated based on item analysis, compositional concept validity, internal consistency, stability, and criterion-related validity using data from 401 midwives. The final version of the scale consisted of 40 items. Cronbach’s α for the overall scale was 0.982. The results for compositional concept validity, internal validity, and criterion-related validity demonstrated that this scale is capable of evaluating a midwife’s practice competencies in intrapartum care. Repeated self-assessment using this scale could improve the competencies of midwives from an early stage, maximize the roles of physicians and midwives, and create an environment that provides high-quality assistance to women.

## 1. Introduction

Midwives and obstetricians are typical healthcare providers with expertise in childbirth. Obstetrician-led care occurs primarily in obstetric units, whereas midwife-led care occurs in freestanding midwifery units (freestanding midwifery units are stand-alone, independent units, while in-hospital midwifery units are hospital based) and home births. Freestanding midwifery units, where women receive midwife-led care, are known to be safe places for women to give birth [1]. A previous study found no difference between midwife- and obstetrician-led care in terms of postpartum hemorrhage, Apgar scores (<7), or neonatal admissions to neonatal intensive care units among low-risk pregnant women and their newborns, confirming that midwife-led care is as safe as obstetrician-led care [1]. Moreover, women who give birth in midwife-led care have expressed higher satisfaction toward childbirth [2,3] and are known to perceive the birthing experience positively. It is also known that a positive birth experience for women strengthens their confidence for future births [4,5]. Midwife-led care offers a sense of safety and satisfaction and increases the rate of vaginal delivery, leading to a reduction in medical costs [4].

In Japan, childbirth at freestanding midwifery units accounted for only 0.5% of all childbirths in 2019 [6]. In England, the percentage of women who give birth at freestanding midwifery units ranges widely, from 4% to 31%, and is increasing [7]. In contrast, it is evident that childbirth in obstetric units is overwhelmingly the norm in Japan. In recent years, however, the Japanese Ministry of Health, Labour, and Welfare has begun promoting in-hospital midwifery units to (i) respond to the diverse pregnancy, childbirth, and childcare needs of parturient women; (ii) address the current shortages of community-based obstetricians and dwindling number of obstetric hospitals and clinics; and (iii) secure safe, secure, and comfortable places for childbirth. In response, the percentage of maternity centers with in-hospital midwifery units in Japan has been increased from 12.0% in 2014 to 16.0% in 2017 [8]. However, training midwives and securing personnel are issues that stand in the way of setting up such midwifery units [9].

Confidence in the professional competence of midwives is related to the amount of practical experience acquired over years of experience [10]. On the other hand, it is also believed that it can be nurtured by providing midwife-led care at freestanding midwifery units. Hunter et al. [11] stated that midwife-led care provides confidence that the birth will proceed normally and to trust the woman’s abilities. They further stated that midwives with experience only in obstetric units may have more difficulty obtaining the necessary confidence to support the woman when providing midwife-led care. Grigg et al. [12] stated that for midwives to provide intrapartum care in freestanding midwifery units, they may need to move away from the medical approach of learning used in obstetric units. In addition, McFarland et al. [13] stated that in integrated maternity practice, midwives have fewer opportunities to provide the high-quality women-centered physiologic care known to benefit childbearing women. Women must be guaranteed the right to choose their own place of birth and to have access to quality support from midwives at all birth locations. It might be necessary for midwives to gain experience in a freestanding midwifery unit to enhance midwife-specific assessments and the technical and care skills needed to provide quality care that trusts the woman’s abilities. However, in a country such as Japan, in which the number of freestanding midwifery units is as small as 10.2% of the total number [14,15], a system that allows midwives to gain experience and confidence in their professional abilities is needed.

Midwives develop clinical expertise toward realizing a desirable, safe, and effective practice through self-reflection on their own practice [16,17]. In midwifery education, an ideal midwifery practice model contributes to the development of midwifery skills, attitudes, behaviors, and trust in natural birth [18]. We hypothesized that self-assessments by midwives in obstetric units based on the practice competencies of midwife-led care in freestanding midwifery units would allow for efficient growth in midwifery practice competencies, including the belief in the normal course of childbirth and care based on trust in a woman’s abilities. Thus, a care practice model based on midwife-led care practices at freestanding midwifery units was developed as a scale that can be used by midwives working at obstetric units to conduct repeated self-assessments, thereby enabling them to efficiently acquire the aforementioned professional midwifery competencies.

Some scales currently exist for assessing midwives’ practice competencies in intrapartum care, such as a scale that assesses the emotional aspects of midwives [19] and an instrument that measures quality of care based on intrapartum care classifications [20]. However, to the best of our knowledge, no existing scales use advanced midwifery at freestanding midwifery units as a model.

Therefore, the purpose of this study was to develop a self-assessment scale for midwifery practice competency based on the characteristics of midwife-led care practices in freestanding midwifery units. 

## 2. Materials and Methods

**Figure 1:**
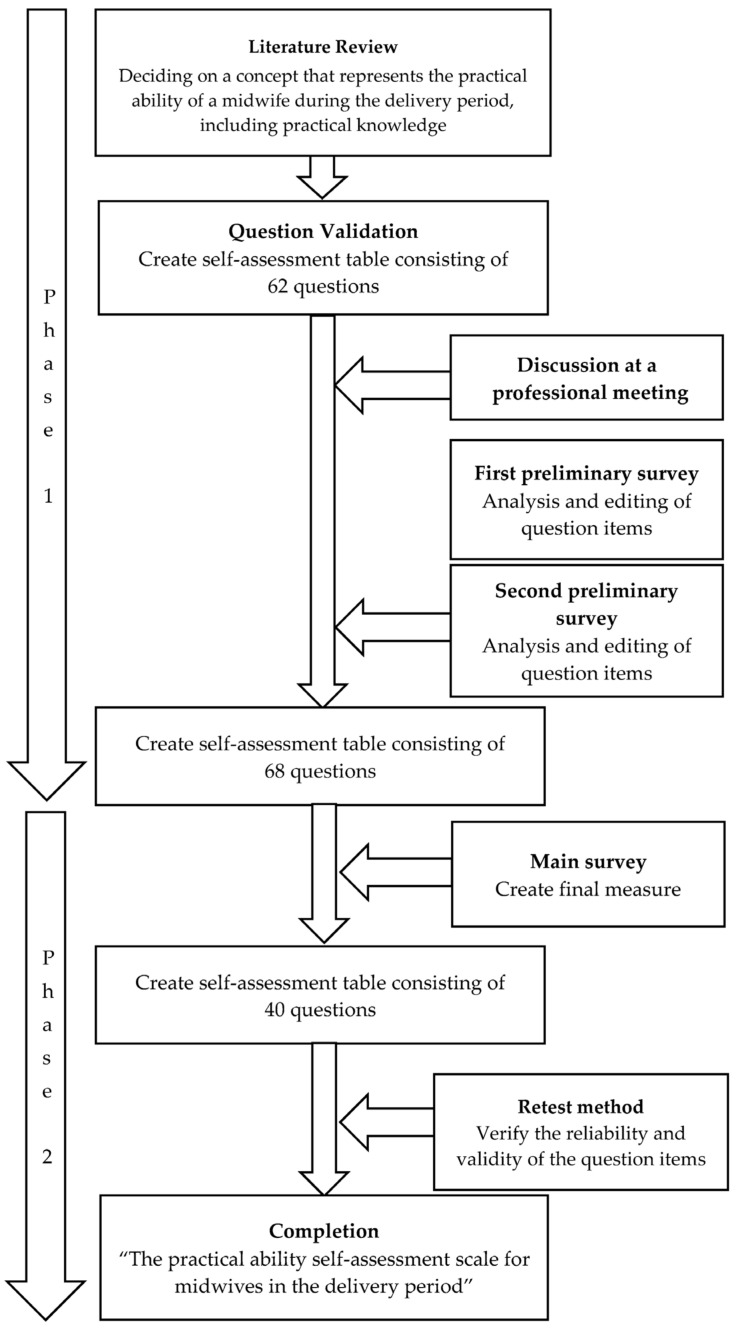
Flow of scale creation.

The present study consisted of two phases. Phase 1 consisted of a literature review, question validation, discussion at a professional meeting, and a preliminary survey conducted at two timepoints. Phase 2 involved verification of the validity and reproducibility of the scale based on the main survey and retest using the questionnaire developed in Phase 1 (see Figure 1).

### 2.1. Phase 1: Item Development and Questionnaire Drafting

Seven sub-concepts and items regarding the construct “High practice competence during the birthing period among Japanese midwives” were extracted from six studies conducted on the subject of midwives with high competence in birth practice in Japan [21,22,23,24,25,26]. We searched the literature published from 2006 to 2016 using the keywords “midwifery”, “clinical competence”, and “childbirth” with the following criterion: original articles that targeted midwives who had worked in freestanding midwifery units for more than 5 years. The search identified five references and one additional reference related to the theme. Subsequently, a meeting involving midwives, two midwifery faculty members, two experts in the field of midwifery, one researcher with experience in scale development, and one co-investigator of this study participated in a meeting to review the validity of the sub-concepts and items. At the meeting, the validity of the question content measuring the constructs, the wording of the questionnaire, and the question items were discussed. The first preliminary survey was conducted on eight faculty members and seven midwives in the field of midwifery to assess the validity of the questions, the ease of answering them, and areas for improvement. A second preliminary survey was conducted on 40 midwives to determine the validity of the questions.

The items used to ascertain the demographics of the participants were age, institution, number of years of experience in birthing assistance, number of assisted births, and whether they were accredited by the Clinical Ladder of Competencies for Midwifery Practice (CLoCMiP) in Japan. These items were presented separately from the items in the scale.

The CLoCMiP level 3 certification system is used to evaluate objectively whether a certain level of midwifery practice competence has been reached, and to examine and certify that the candidate has reached CLoCMiP level 3. Only midwives meeting a set number of requirements, such as attending mandatory training, participating in a specific number of assisted births, and performing primary cases, can apply for certification. After applying, midwives must take an examination. If they pass, they receive CLoCMiP level 3 certification. A midwife certified at CLoCMiP level 3 is recognized as having the practical ability to provide autonomous care at an in-hospital midwifery unit [27] and assume a leadership role for other midwives in the midwifery outpatient setting. This certification is renewable every 5 years.

### 2.2. Phase 2: Evaluation of the Scale’s Validity and Reproducibility

#### 2.2.1. Survey

The scale’s main survey and retest were conducted on 1181 midwives from 65 childbirth facilities throughout Japan between September 2017 and March 2018. The nursing administrators and affiliated midwives from the candidate institutions cooperating with the study were provided a written explanation of the study, including information on the objectives and significance of the study, the voluntary nature of their participation, and the methods, data handling, publication of results, and contact information. The survey was an anonymous, self-administered questionnaire. Returning a completed survey in a sealed envelope was considered to indicate consent to participate.

#### 2.2.2. Survey Validity and Stability Confirmation (Statistical Analysis)

##### Item Analysis

Correlations between questionnaire items and scale scores were determined to develop a more accurate scale with a lower limit of the item-total correlation was 0.6 ≤ γ.The normality of the items was verified, and the reliability of the items was analyzed based on ceiling and floor effects as well as item-total correlations.

##### Compositional Concept Validity

Exploratory factor analysis with promax rotation, which can emphasize the merit between items, was used to examine the scale’s factor structure based on the relationships among multiple variables. The analysis conditions were as follows: the fixed value of the factor was ≥1, the item’s factor loadings were ≥0.4, and no multiple factor loadings were >0.4.

##### Internal Consistency

This study aimed for a scale with a Cronbach’s α coefficient ≥ 0.85 to indicate acceptable internal consistency.

##### Stability

The intra-class correlations and Pearson’s correlation coefficients for the subscale and total scale scores were determined, and reproducibility was examined after the first response and again after 1 month to confirm stability. The timing of the retest was set for 1 month following the initial survey, when the memory of their initial responses was expected to have faded and the midwife’s experience of childbirth care was considered less likely to affect her ability to practice.

##### Criterion-Related Validity

Criterion-related validity is typically confirmed by correlations between existing and new scales; however, no existing scales were able to measure care competency equivalent to “midwife-led care in freestanding midwifery units”. Therefore, we conducted a good–poor (G–P) analysis on the following items: whether midwives had CLoCMiP level 3 certification, the number of years of experience in birth practice, and the number of times involved in a birth. The G–P analysis was performed by quadrating the scale’s total points to assess whether it could indicate a high level of practice competence, and then making comparisons between midwives in the 25th percentile or lower and in the 75th percentile or higher. Statistical calculations were performed using SPSS 25 (IBM SPSS Statistics 25.0; IBM Corp, Armonk, NY, USA).

##### Ethics

This study was approved by the Ethics Committee on Epidemiological Studies at Kagoshima University on 15 January 2020 (approval no. 179994 (371) epidemiology—revision 5), and was conducted according to the guidelines of the Declaration of Helsinki.

This work was supported by JSPS KAKENHI Grant Number 18K10467. The funding body was not involved in the design of the study, the collection, analysis, and interpretation of data, or in the preparation of the manuscript.

## 3. Results

### 3.1. Phase 1: Item Development and Questionnaire Drafting

#### 3.1.1. Literature Review and Question Validation

The sub-concepts of this scale determined that the following seven components of midwifery decision-making would be used from the literature [21]: “determining the normality and stage of labor”, “respecting and understanding the wishes of the woman”, “ensuring the woman’s self-determination”, “understanding and caring for the stability of the family”, “caring for the physical and psychological needs of the woman”, “ consideration of potential risks and ensuring the safety of the mother and child”, and “integrating the midwife’s knowledge and beliefs”. To produce the characteristics of practical skills, questionnaire items were formulated with reference to previous papers on maternal care [22,23,24,25,26]. A total of 62 items were generated.

#### 3.1.2. Discussion at a Professional Meeting

After a discussion about items at the meeting regarding the validity of the questions, the question wording, and the question items, the number of questions was reduced to 54.

#### 3.1.3. First Preliminary Survey

The results of the first preliminary survey showed that some of the questions did not present content appropriate to the situation of the facility and that additional questions needed to be added. Furthermore, because of the bias in the responses to the options, the questionnaire was changed from one that measured psychological distance to one that asked about the degree of conformity with reality. As a result, a 68-item scale was developed (see Supplementary Materials, Table S1).

#### 3.1.4. Second Preliminary Survey

A second preliminary survey was conducted to determine the validity of the scale using 68 questions. After item-total correlation analysis of the data, all 68 questions were used because the lowest correlation coefficient was 0.435.

### 3.2. Phase 2: Evaluation of Tool’s Validity and Reproducibility

#### 3.2.1. Survey Participants

In total, 65 Japanese maternity centers, comprising 1181 midwives, agreed to cooperate with this study. Of these midwives, 401 (33.9%) provided valid responses. The low number of valid responses may be due to the fact that when we asked for cooperation in this survey, we also asked for cooperation in the retest at the same time, which the participants may have felt was a burden. Factor analysis requires a ratio of 5 to 10 persons per variable, and because the number of items in this study was 68 and the minimum sample size was 340 persons, we determined that this was an appropriate sample size for analysis [28]. The mode age group of the participants was 30–39 years, and the mean ± standard deviation (SD) of the amount of experience providing birthing assistance and number of birth assistance cases were 10.57 ± 9.81 years and 342.07 ± 481.22 cases, respectively. Additionally, 184 (45.9%) midwives had CLoCMiP level 3 certification. Hospitals were the most common affiliation, reported by 331 respondents (82.5%; see Table 1). Most of the target maternity centers were located in urban areas, and hospitals were primarily general hospitals. In addition, there were also no clinics or freestanding midwifery units located more than 1 h from an obstetric care facility.

#### 3.2.2. Confirmation of Survey Validity and Reproducibility

##### Item Analysis

The item analysis showed a normal distribution, and the α coefficient was as high as 0.754 for all items, which did not lead to item deletion. Although no items displayed floor effects, one item with a mean ceiling effect was deleted from sub-concept VII. Additionally, 12 items with item correlations > 0.8 were considered to measure the same content and thus were merged into a single item with a high corrected item-total correlation. Therefore, 13 items were removed in total.

##### Compositional Concept Validity

Based on the item analysis results, a factor analysis for the 55 items with an eigenvalue ≥ 1 confirmed that there were four factors; thus, a further factor analysis was repeated with the number of factors set to four. Additionally, we removed items showing high factor loadings (<0.400 or ≥0.400 for multiple factors). Ultimately, 15 items were removed, leaving 40 items in the midwifery practice competency self-assessment scale; these items were designated as midwifery practice competencies in the birth process (see Supplementary Materials, Table S2).

Factor I, “Care that respects the birth wishes of the parturient woman and her family,” consisted of 15 items, including “I can ascertain what kind of hopes the woman has for her birth”, “I can capture the thoughts of the parturient woman based on her wishes for birth”, “I can devise care that incorporates the wishes of the parturient woman”, and “I can care for the parturient woman while respecting her decision-making”. These were based on the ability to respect parturient women’s wishes and decisions. Factor II, “Ability to make decisions and respond to sudden emergencies with a good understanding of the scope of responsibility,” consisted of nine items, including “I can perform postpartum maternal hemorrhage management” and “I can monitor the progress of delivery and plan the timing of action in concert with the doctor”, and was based on midwives’ ability to prepare themselves and their supplies daily in case of an emergency, and the ability to make decisions and respond to such emergencies in parturient women or newborns within the scope of their responsibility while using knowledge gained through experience. Factor III, “Care tailored to the changes of the parturient woman and the condition of both the mother and child,” also consisted of nine items, including “I can effectively relieve birthing pains using the labor relaxation method”, “I can manage the woman’s physical condition according to the progress of birth”, “I can choose the labor relaxation method according to the parturient woman’s condition”, and “I can provide care that matches the natural movements of the birthing woman”. These were based on the ability to assist in childbirth while responding to physical and mental changes in the parturient woman as the birth progressed, providing the woman with peace of mind, and supporting the movement of mother and child in accordance with the mechanisms of natural childbirth. Factor IV, “Ability to gather information and make accurate, comprehensive decisions,” consisted of seven items, including “I can select the necessary information for predicting the course of birth according to its progress”, “I can judge the health of the fetus by integrating cardiotocography and other information”, “I can accurately judge the condition of the parturient woman”, “I can predict the course of the birth from an assessment of basic information about the parturient woman”, and “I can select the necessary information for predicting the course of birth according to its progress”. These were based on the ability to collect basic and other types of information using the five senses, assess the psychological and physiological conditions of the mother and child, and make comprehensive and accurate judgments in accordance with the data.

The mean ± SD for the total score of the scale was 143.37 ± 24.88. The cutoff score for this scale was measured at 156 with receiver operator characteristic (ROC) curves using the number of years of experience in birthing assistance and the number of assisted births.

Furthermore, the correlation coefficients between the four factors ranged from 0.682 to 0.793, indicating overall positive correlations. Factors I and III (r = 0.793), Factors II and III (r = 0.786), Factors II and IV (r = 0.750), and Factors III and IV (r = 0.770) were found to have strong correlations, while the other factors were found to have only moderate correlations (see Supplementary Materials, Table S2).

##### Internal Consistency

Cronbach’s α was 0.982 for the overall scale, and 0.957, 0.953, 0.959, and 0.936 for Factors I–IV, respectively.

##### Stability

The intra-class correlation coefficients for the test–retest scores ranged from 0.699 to 0.789 for the subscales, with Factor IV being the lowest (r = 0.699) and Factor II being the highest (r = 0.789). The overall scale had a correlation coefficient of r = 0.794 and a Pearson’s correlation coefficient of r = 0.795 (see Table 2).

##### Criterion-Related Validity

The G–P analysis was conducted for the total score assessing midwifery practice competence to determine the scale’s validity: namely, CLoCMiP level 3 certification, years of midwifery experience, and number of births. The total score was divided into quadrants, and comparisons were made for midwives below the 25th and above the 75th percentiles. The results showed significant differences for all items (see Table 3).

##### Completion of the Self-Assessment Scale for Midwives’ Practice

The scale comprised four factors and 40 items describing the characteristics of midwifery competency. All items were scored on a 5-point Likert scale (1 = hardly ever, 2 = sometimes, 3 = often, 4 = usually, 5 = always).

## 4. Discussion

The scale in this study was developed after a literature review on advanced-level midwife-led care at freestanding midwifery units in Japan. The validity of the scale was confirmed through the following: evaluation of content validity by experts, construct validity through factor analysis, evaluation with Cronbach’s α coefficients, testing stability using the intra-class correlation coefficients at the beginning and after 1 month, and testing reproducibility using Pearson’s correlation coefficients. Criterion-related validity was confirmed by conducting a G–P analysis of the following: attainment of a highly-autonomous advanced-level certification, number of years’ experience in providing birthing assistance, and the number of assisted deliveries.

The present scale is composed of four factors. When the correlation of factors was analyzed, a strong correlation was found between Factor I and Factor III (r = 0.793), Factor II and Factor III (r = 0.786), Factor II and Factor IV (r = 0.750), and Factor III and Factor IV (r = 0.770). All other factors were found to have a moderate correlation. Cronbach’s α coefficient for the overall scale was 0.982, and that between each of the factors was 0.957 for Factor I, 0.953 for Factor II, 0.959 for Factor III, and 0.936 for Factor IV. The present scale showed good reliability, indicating that it is a good scale for conducting self-assessments of the practice competencies of midwives, which are constructed based on advanced-level midwife-led care.

Factor I include general competencies necessary for midwifery practice as indicated by the International Confederation of Midwives (ICM): 1. e, protect the basic rights of individuals when providing midwifery care; 1. g, facilitate women’s individual choices about their care; and 1. h, demonstrate effective interpersonal communication with women and families [29]. It also included a higher practice competence section on nonverbal communication that takes the individuality and needs of women into account. Women want woman-centered care based on a close relationship with midwives [30]. It is known that verbal and nonverbal communication with health-care providers such as midwives and women-focused individualized midwifery care are linked with women’s satisfaction with childbirth [31,32]. Care respecting woman’s decision-making addresses important aspects of women-centered care and is also believed to have a significant impact on women’s satisfaction with childbirth [33]. Furthermore, to provide good quality shared decision-making in intrapartum care, the women’s expectations and preferences need to be explored, and their consent must be obtained [34]. As evidenced by the above, the factor “Care that respects the birth wishes of the parturient woman and her family” contains the following aspect of advanced-level midwife-led care: utilizing strong communication skills with the women and the ability to undertake shared decision-making.

Factor II includes the competencies needed for midwifery practice as outlined by the ICM: (a) General competencies: “1. f Adhere to jurisdictional laws, regulatory requirements, and codes of conduct for midwifery practice”, “1. h Demonstrate effective interpersonal communication with women and families, health-care teams, and community groups”, “1. j Assess the health status, screen for health risks, and promote the general health and well-being of women and infants”, and “1. l Recognize conditions outside the midwifery scope of practice and make referrals appropriate” and (b) care during labor and immediately after birth: “preventing complications” in “3. b Manage a safe spontaneous vaginal birth and prevent complications” and “3. c Provide care of the newborn immediately after birth” [29]. However, the present scale includes items on high practice competence such as those required in responding to emergencies. The items in this factor incorporate the basic emergency response competencies of a midwife. Apparently, despite having knowledge about postpartum hemorrhage and neonatal resuscitation, many midwives lack practical experience and require training [35,36]. These were included as items as they are the aspects of care that midwives must be able to practice for advanced-level midwife-led care. The abilities to provide primary treatment and emergency responses as needed, which are considered to be indispensable competencies for providing high-quality midwifery care, were included [37]. Women who needed to be transferred from a maternity unit to an obstetric unit during labor accounted for 12–15% of all deliveries [38]. Therefore, advanced-level midwife-led care in which the midwife acts “while considering the scope of responsibility of a midwife” and “in concert with the doctor” is essential for providing autonomous midwifery care. As evidenced by the above, the factor “Ability to make decisions and respond to sudden emergencies with a good understanding of the scope of responsibility” contains the following aspect of advanced-level midwife-led care: the ability to respond to sudden changes that may occur in the woman and her newborn, and the ability to decide to consult a physician before putting the life of a woman’s child in danger.

Factor III included the competencies for care during labor and immediately after birth outlined by the ICM: “safe and natural vaginal birth management” consisting of “3. a Promote physiologic labor and birth” and “3. b Manage a safe spontaneous vaginal birth; prevent and detect complications” [29]. However, the present tool includes items on high practice competence that consider a midwife’s ability to observe and assess the conditions of the woman and her child and provide care accordingly. Pain relief is the most important element of the childbirth experience for a woman who has just given birth [39]. Therefore, midwives address the effectiveness of their pain control methods and attempt to lessen the pain experienced by the woman by adopting different positions [40]. This highlights the fact that midwives consider elements such as (i) respect for the natural process, (ii) assistance according to the woman’s wishes, and (iii) food as care as an important concept of the midwifery processes when providing care [41]. As evidenced by the above, the factor “Care tailored to the changes of the parturient woman and the condition of both the mother and child” contains the following aspect of advanced-level midwife-led care: helping to alleviate a woman’s sensation of pain and setting up an environment that prepares a woman’s body and mind for childbirth.

Factor IV includes the competencies needed for midwifery practice outlined by the ICM: “3. a Promote physiologic labor and birth” and “3. b Manage a safe spontaneous vaginal birth and prevent complications” [29]. However, the present scale includes items on high practice competence that assess the integrative skills that midwives develop through experience. The safety of childbirth ensured through technical expertise and the health-care system increase a women’s satisfaction with maternity care [42]. Furthermore, midwives use experience-based knowledge such as “touch” and “reading of body language” as clues for making decisions [43]. As evidenced by the above, the factor “Ability to gather information and make accurate, comprehensive decisions” contains the following aspect of advanced-level midwife-led care: comprehensive decision-making skills developed through midwifery experience.

The structure of the present tool begins with the woman-centered care characteristic of midwife-led care followed by the ability to respond in order to protect the safety of the mother and child, physiological care that draws on the natural power of the midwife’s female body, and comprehensive judgment developed through the midwife’s use of her five senses and experience. Sigridur et al. [44] indicated five elements of professionalism among good midwives: professional care provision, professional wisdom, the midwife’s development, interpersonal competence, and professional competence. The midwife’s professional care is at the core of midwifery while caring for women and their families within the professional sphere. The professional wisdom of the midwife develops through the interplay of knowledge and experience. The midwife’s professional competence demonstrates competence in ensuring the safety of the mother and child. The interpersonal competence of midwives empowers communication. The midwife’s development consists of knowing and nurturing the self. Contrasting the item content of the present scale with these factors, Factor I of the scale is caring and the midwife’s interpersonal competence, Factor II is professional competence, Factor III is professional wisdom and professional competence, and Factor IV is professional wisdom, which covers all four factors. The present scale was developed using midwife-led care practices and reflects good midwifery professionalism.

In the maternity unit, midwives are frequently placed in unenviable positions of relative powerlessness and are sometimes presented with a conflict between a drive to agree with authority and supporting the safe, evidence-based choices of the childbearing women in their care [45]. Midwives in the maternity ward also need to be professional and capable of asserting their own views as midwives to protect women from a professional standpoint. We believe that midwives’ assertion of their opinions to protect women will promote understanding of the expertise of midwives among obstetricians and other professionals with whom they work and help create an environment surrounding childbirth that takes advantage of the expertise of each profession. We believe that midwives working in obstetric units should use the present scale for reflection and for improving awareness of their own expertise so that as midwives, they can effectively gain confidence in the normal course of delivery and trust in the abilities of women. Although this scale does not include growth as a midwife, which is one component of a good midwifery professional, we believe that the accumulation of successive reflections on one’s practice using this scale will lead to growth as a midwife.

The present scale not only covers the basic competencies needed by midwives at all levels of experience, from new to advanced, but also the high-quality care and professionalism needed to be an even better midwife. We believe that by using this scale to reflect on their own practice even before graduation, midwifery students will be able to receive training with an awareness of professionalism starting from their basic midwifery education years.

The present scale was developed to improve midwives’ capacity for midwife-led care, but it also reflects the professionalism of a good midwife. We believe that by using this tool for self-assessment of birthing care, midwife-led care competence can be acquired efficiently and the assessment items in this scale can be used as a growth model.

The present scale needs to be refined further, such as reconsideration of cutoff score, in the future in the course of validation with a higher number of subjects. In addition, the competencies in this scale reflect the current situation and may need to be revised in the future in accordance with any changes in social conditions to the medical system.

## 5. Conclusions

The present tool covers the competencies required by midwives, and the items serve as a practice model of the skills, attitudes, and behaviors of advanced-level midwives who provide midwife-led care at freestanding midwifery units. It can be used to evaluate the practice competencies of midwives involved in intrapartum care as proven by the compositional concept validity, internal validity, and criterion-related validity tests. In addition, the effectiveness of the present scale for evaluating practical skills targeting a large number of midwives needs to be validated.

## Figures and Tables

**Table 1:** Characteristics of the survey respondents (N = 401).

Characteristic		N (%)
Age (years)	20–29	112 (27.9)
	30–39	119 (29.7)
	40–49	107 (26.7)
	50–59	55 (13.7)
	≥60	7 (1.7)
	No answer	1 (0.2)
Institution	Hospital	331 (82.5)
	Clinic	50 (12.5)
	freestanding midwifery units	12 (3.0)
	No answer	8 (2.0)
Experience in birthing assistance (years)	mean ± SD	10.57 ± 9.81
	<5	118 (29.4)
	5–10	119 (29.7)
	11–20	118 (29.4)
	21–30	40 (9.9)
	≥31	6 (1.5)
CLoCMiP level 3 certification	Advanced midwife	184 (45.9)
	Non-advanced midwife	217 (54.1)

SD: standard deviation; CLoCMiP: Clinical Ladder of Competencies for Midwifery Practice. CLoCMiP level 3 is regarded as proof that a midwife possesses a high level of practical ability to perform autonomous midwifery activities. Advanced midwife: Midwife certified as CLoCMiP level 3.

**Table 2:** Correlation of questionnaire scores by the test–retest method (N = 114).

Item	Score (mean ± SD) Title 3		Intra-Class Correlation Coefficient	
	1st	2nd	Correlation Coefficient	95% Confidence Interval
Factor I	56.52 ± 8.80	55.52 ± 8.98	0.702 ***	0.596–0.784
Factor II	33.33 ± 6.67	33.02 ± 6.33	0.789 ***	0.708–0.749
Factor III	32.63 ± 6.12	32.39 ± 5.71	0.757 ***	0.666–0.825
Factor IV	26.16 ± 4.49	25.71 ± 4.44	0.699 ***	0.591–0.782
Total score	148.64 ± 23.68	146.64 ± 23.70	0.794 ***	0.715–0.853

*** *p* < 0.001; one-way analysis of variance. SD: standard deviation.

**Table 3:** Examination of validity using good–poor analysis.

Item	Midwives under 25% of Total Scale (n = 101)	Midwives over 75% of Total Scale (n = 115)	*p*
Total score	110.15 ± 15.8 ^a^	169.82 ± 11.3	<0.001
Advanced midwife	22 (21.8%) ^b^	78 (67.8%)	<0.001
Experience in birthing assistance (years)	6.10 ± 6.22 ^a^	14.36 ± 7.75	<0.001
Number of assisted births	145.87 ± 212.60 ^a^	554.09 ± 638.50	<0.001

Data are expressed as number (%) or mean ± standard deviation. ^a^ unpaired *t*-test. ^b^ chi-squared test.

## Data Availability

The datasets generated during the present study are available from the corresponding author on reasonable request.

## Supplementary Materials

The following supporting information can be downloaded at: https://www.mdpi.com/article/10.3390/ijerph20031859/s1, Table S1: instrument draft with 68 items; Table S2: the standard-related validity of the intrapartum period practical midwifery skill self-rating scale.
