# Supplementary material for: Development and Validity of an Intrapartum Self-Assessment Scale Aimed at Instilling Midwife-Led Care Competencies Used at Freestanding Midwifery Units
Source: Int J Environ Res Public Health. 2023 Jan 19;20(3):1859. doi: 10.3390/ijerph20031859 (PMC9914374; doi:10.3390/ijerph20031859)
Supplement: Supplementary file 1 [file ijerph-20-01859-s001.zip › ijerph-2085695-supplementary.pdf]

Supplementary Materials

Table S1. Instrument draft with 68 items.

Sub-concepts		Item
I	1.	I can predict the course of the birth from an assessment of basic information about the parturient woman.
	2.	I can predict the course of the birth while considering the psychological state of the parturient mother.
	3.	I can select the necessary information for predicting the course of birth according to its progress.
	4.	I can accurately observe the conditions of the parturient woman and fetus using my five senses.
	5.	I can accurately judge parturient progress based on the information I gather using my five senses.*
	6.	I can judge the health of the fetus by integrating cardiotocography and other information.
	7.	I can judge whether the birth process is normal, considering the passage of time as well.
	8.	I can accurately determine the health of the fetus.*
	9.	I can accurately judge the condition of the parturient woman.
	10.	I can accurately predict the parturient progress.
II	11.	I can understand the character of the parturient woman through communication with her.
	12.	I can understand the relationship between the parturient woman and her family by observing their communication.
	13.	I can ascertain the parturient woman's feelings about her previous birth experience in the case of a multipara.
	14.	I can ascertain what kind of hopes the woman has for her birth.
	15.	I can capture the thoughts of the parturient woman based on her wishes for birth.
	16.	I can devise care that incorporates the wishes of the parturient woman.
	17.	I can make sure how the parturient woman feels about her current condition.
	18.	I can support the physical and emotional reactions of parturient woman as they are, without hindering their expression.
III	19.	I can provide the necessary information for the birth mother to make decisions.*
	20.	I can support decision-making while presenting options to the parturient woman.*
	21.	I can assist the decision-making of the parturient woman according to her personality.
	22.	I can care for the parturient woman while respecting her decision-making.
	23.	I can be an advocate for the parturient woman encouraging communication of her thoughts to others.
	24.	I can regulate the human environment that interferes with the decision-making of the birth mother.
	25.	I can provide care while confirming the feelings of the parturient woman during birth.
	26.	If a change in decision-making is required, I can explain the need for the change and assist in making another decision.

IV	27.	I can care for the parturient woman and her partner/family according to their relationships.
	28.	I can sense the situation of the birth mother and her family from the atmosphere in the room.
	29.	I can support their involvement in childbirth, depending on their situation.
	30.	I can create an environment where the family and the birth mother can be together during the delivery.
	31.	I can take care of communication with the parturient woman and her family.
	32.	I can take care of the health of my family members accompanying me to the birth.
	33.	I can explain things so that the family of parturient woman can also understand the progress.
	34.	I can help birth mothers and their families feel united in giving birth.
V	35.	I can condition the woman's body according to the progress of birth.
	36.	I can adjust the environment of the maternity room according to the condition of the parturient woman.
	37.	I can choose to undertake appropriate techniques to facilitate birth according to its progress.
	38.	I can choose the labor relaxation method according to the parturient woman's condition.
	39.	I can effectively facilitate birth using appropriate non-pharmacological techniques.
	40.	I can effectively relieve birthing pains using the labor relaxation method.
	41.	I can provide care that matches the natural movements of the birthing woman.
	42.	I can manage the woman's physical condition according to the progress of birth.
	43.	I can provide care that brings out the independence of the mother.*
	44.	I can assist in the birth using the functions of the mother and child according to their conditions.
	45.	I can minimize and prevent harm to the mother by using the techniques of assisted birth.*
	46.	I can provide treatment according to the condition of the newborn immediately after birth.
	47.	I can provide support for attachment formation according to the conditions of the mother and child immediately after delivery.
	48.	I can support the initiation of breastfeeding according to the conditions of the mother and child immediately after delivery.
VI	49.	I can detect early signs of abnormalities in the mother and child.*
	50.	I can accurately judge the risk of abnormalities according to the progress of the birth.
	51.	I can accurately determine when to intervene with the necessary measures to avert a crisis.*
	52.	I can simulate in concrete terms what I would do in an emergency.
	53.	I can make sure I have the supplies needed for the procedure in the case of an emergency.
	54.	I can check the status of the medical staff in advance in the case of an emergency.*
	55.	I can act while considering the scope of responsibility of a midwife.
	56.	I can perform postpartum maternal hemorrhage management.

	57.	I can perform neonatal resuscitation.
	58.	I can monitor the progress of delivery and plan the timing of action in concert with the doctor.
VII	59.	I can use the knowledge I have gained from my own past experiences to determine parturient progress.*
	60.	I can use the skills I have developed in my experience.*
	61.	I can use empirical knowledge.
	62.	I can think of birthing based on my own values.
	63.	I can consider care based on my own values.*
	64.	I can establish a trusting relationship with the parturient woman.
	65.	I can establish a trusting relationship with doctors.
	66.	I can communicate well with other professions.
	67.	I can reflect on the birth with the woman after the birth.
	68.	I can reflect on my own midwifery practice after the birth.

* This item was deleted.

Table S2. The standard-related validity of the intrapartum period practical midwifery skill self-rating scale.

Item	Component			
	**Facto r I**	**Facto r II**	**Facto r III**	**Facto r IV**
**Factor I: Care that respects the birth wishes of the parturient woman and her family (α = 0.957)**				
1. I can ascertain what kind of hopes the woman has for her birth.	0.900	0.055	–0.321	0.121
2. I can ascertain the parturient woman's feelings about her previous birth experience in the case of a multipara.	0.870	0.066	–0.228	–0.066
3. I can capture the thoughts of the parturient woman based on her wishes for birth.	0.830	–0.024	–0.074	0.097
4. I can make sure how the parturient woman feels about her current condition.	0.774	–0.085	0.130	–0.009
5. I can devise care that incorporates the wishes of the parturient woman.	0.772	–0.075	0.051	0.044
6. I can provide care while confirming the feelings of the parturient woman during birth.	0.690	–0.094	0.183	0.052
7. I can understand the character of the parturient woman through communication with her.	0.654	0.083	0.080	–0.075
8. I can understand the relationship between the parturient woman and her family by observing their communication.	0.652	0.072	–0.001	–0.060
9. I can assist the decision-making of the parturient woman according to her personality.	0.617	0.081	0.254	–0.043
10. I can care for the parturient woman while respecting her decision-making.	0.601	–0.007	0.156	0.089
11. I can support decision-making while presenting options to the parturient woman.	0.575	0.133	0.165	0.037
12. I can be an advocate for the parturient woman encouraging communication of her thoughts to others.	0.557	0.090	0.290	–0.145
13. I can care for the parturient woman and her partner/family according to their relationships.	0.545	–0.060	0.316	0.057
14. I can take care of communication with the parturient woman and her family.	0.495	–0.025	0.236	0.107
15. If a change in decision-making is required, I can explain the need for the change and assist in making another decision.	0.481	0.159	0.233	–0.004
**Factor II: Ability to gather information and make accurate, comprehensive decisions (α = .953)**				
16. I can perform postpartum maternal hemorrhage management.	–0.060	0.968	–0.045	0.022
17. I can perform neonatal resuscitation.	–0.007	0.881	–0.086	–0.045
18. I can make sure I have the supplies needed for the procedure in the case of an emergency.	0.046	0.843	–0.071	0.021
19. I can act while considering the scope of	0.115	0.791	0.061	–0.099

responsibility of a midwife.				
20. I can simulate in concrete terms what I would do in an emergency.	0.042	0.762	0.106	−0.041
21. I can monitor the progress of delivery and plan the timing of action in concert with the doctor.	0.004	0.678	0.083	0.141
22. I can accurately judge the risk of abnormalities according to the progress of the birth.	0.021	0.619	0.222	0.065
23. I can provide treatment according to the condition of the newborn immediately after birth.	0.022	0.574	0.211	0.021
24. I can use empirical knowledge.	0.030	0.508	0.184	0.145
**Factor III: Care tailored to the changes of the parturient woman and the condition of both the mother and child (α = .959)**				
25. I can effectively relieve birthing pains using the labor relaxation method.	−0.042	−0.048	0.987	−0.069
26. I can manage the woman's physical condition according to the progress of birth	0.103	0.006	0.779	0.010
27. I can choose the labor relaxation method according to the parturient woman's condition.	−0.020	0.061	0.761	0.086
28. I can effectively facilitate birth using appropriate non-pharmacological techniques.	−0.074	0.114	0.740	0.086
29. I can provide care that matches the natural movements of the birthing woman.	0.111	0.120	0.724	−0.074
30. I can provide care according to the psychological state of the parturient woman.	0.160	0.039	0.709	−0.013
31. I can choose to undertake appropriate techniques to facilitate birth according to its progress.	−0.015	0.110	0.697	0.113
32. I can assist in the birth using the functions of the mother and child according to their conditions.	−0.003	0.221	0.630	0.048
33. I can adjust the environment of the maternity room according to the condition of the parturient woman.	0.174	0.033	0.598	0.043
**Factor IV: Ability to gather information and make accurate, comprehensive decisions (α = 936)**				
34. I can predict the course of the birth from an assessment of basic information about the parturient woman.	−0.032	−0.029	−0.044	0.934
35. I can predict the course of the birth while considering the psychological state of the parturient mother.	0.056	−0.084	0.060	0.803
36. I can select the necessary information for predicting the course of birth according to its progress.	−0.048	0.019	0.153	0.778
37. I can judge the health of the fetus by integrating cardiotocography and other information.	0.093	0.267	−0.231	0.655
38. I can judge whether the birth process is normal, considering the passage of time as well.	0.012	0.262	0.139	0.506
39. I can accurately observe the conditions of the parturient woman and fetal using my five senses.	0.076	0.067	0.270	0.494
40. I can accurately judge the condition of the parturient woman.	0.135	0.142	0.151	0.461

Correlation between factors: Factor II	0.682		
Factor III	0.793	0.786	
Factor IV	0.696	0.750	0.770

All Cronbach's α = 0.982;

Factor extraction method: main factor method (promax rotation).
